# Antioxidant and Renoprotective Effects of *Spirogyra neglecta* (Hassall) Kützing Extract in Experimental Type 2 Diabetic Rats

**DOI:** 10.1155/2013/820786

**Published:** 2013-06-03

**Authors:** Atcharaporn Ontawong, Naruwan Saowakon, Pornpun Vivithanaporn, Anchalee Pongchaidecha, Narissara Lailerd, Doungporn Amornlerdpison, Anusorn Lungkaphin, Chutima Srimaroeng

**Affiliations:** ^1^Department of Physiology, Faculty of Medicine, Chiang Mai University, Chiang Mai 50200, Thailand; ^2^Division of Physiology, School of Medical Sciences, University of Phayao, Phayao 56000, Thailand; ^3^School of Biology, Institute of Science, Suranaree University of Technology, Nakhon Ratchasima 30000, Thailand; ^4^Department of Pharmacology, Faculty of Science, Mahidol University, Bangkok 10400, Thailand; ^5^Faculty of Fisheries Technology and Aquatic Resources, Maejo University, Chiang Mai 50290, Thailand

## Abstract

*Spirogyra neglecta* extract (SNE) has shown antihyperglycemia and antihyperlipidemia in type 2 diabetic mellitus (T2DM) rats. This study investigated the antioxidant and renoprotective effects of SNE in T2DM rats induced by high-fat diet with low-single dose streptozotocin. T2DM rats were fed daily with SNE (0.25, 0.5, and 1 g/kg BW) for 12 weeks. Renal morphology, malondialdehyde levels, qPCR, and western blotting were analyzed. Renal cortical slices were used to determine renal transport of organic anions, which are estrone sulfate and *para*-aminohippurate, mediated through organic anion transporter 3-Oat3. Insulin and PKC*ζ* were known to activate Oat3 function while it was inhibited by PKC*α*. Compared to T2DM, plasma glucose, triglyceride, insulin resistance, renal morphology, and malondialdehyde levels were significantly improved by SNE supplementation. Reduced glutathione peroxidase and nuclear factor *κ*B expressions were related to antioxidant effect of SNE. Oat3 mRNA and protein were not different among groups, but insulin-stimulated rOat3 followed by anion uptakes was abolished in T2DM. This was restored in the slices from SNE treatment. The mechanism of SNE-improved Oat3 was associated with PKC*α* and PKC*ζ* expressions and activities. These findings indicate that SNE has beneficial effects on renal transport through antioxidant enzymes and PKCs in T2DM rats.

## 1. Introduction

*Spirogyra neglecta* (SN) has been widely grown in the Nan River, North of Thailand. It is commonly used as an ingredient in several Northern Thai food and contains high amount of nutritional compositions, including basic nutrients, which are carbohydrate, fat, proteins, multivitamins, and mineral substances [1, 2]. Previous *in vivo* studies have indicated that this species has antigastric ulcer and anti-inflammatory effects in rats [3]. Antioxidant activity of crude SN extract (SNE) has also been demonstrated and suggested that SNE may subsequently be used as cosmeceutical and nutraceutical products [4]. More recently, SNE at the dose of 1.0 g/kg BW was able to reduce plasma glucose, triglyceride, and free fatty acid in type 2 diabetic mellitus (T2DM) rats [5]. 

Diabetes mellitus is a common disease that currently affects nearly 350 million people worldwide [6]. T2DM is mostly associated with the combination of hyperglycemia, insulin resistance, and/or relative insulin deficiency [7]. Uncontrolled hyperglycemia resulting from long-term rising blood glucose levels leads to development of diabetes complications, including retinopathy, peripheral neuropathy, and nephropathy [8]. Recently, the excessive production of reactive oxygen species (ROS) that leads to oxidative stress has been suggested to participate in diabetes-associated nephropathy and the mechanisms of hyperglycemia-induced ROS production in diabetic condition have been proposed [9, 10]. The overproduction of ROS in the kidney activates stress-activated signaling molecules, including NF*κ*B, protein kinase C (PKC), mitogen-activated protein kinase (MAPK), and advanced glycation end products (AGE) by increasing hydrogen peroxide in mesangial cells and causing lipid peroxidation in glomeruli [11–13]. 

Kidney plays an important role in the elimination of several compounds, including drugs, toxins, and endogenous substances. The active secretion of cationic and anionic compounds to the tubular lumen appears to be restricted to the basolateral membrane of renal tubules by basolateral membrane transporters [14]. At the present, several renal membrane transporters have been cloned and identified [15], and among these, organic anion transporter 3 (Oat3: SLC22A8) is highly expressed [16] and plays a major role in cellular uptake of organic anions across the basolateral membrane of renal proximal tubules. As Oat3 knockout mouse model conclusively demonstrated that Oat3 also transports organic anions in liver and choroid plexus [17]; thus, its positioning has a major impact on the excretion of anionic drugs and xenobiotics for controlling systemic homeostasis. These organic anions were (1) endogenous, for example, prostaglandins E2 and F2*α*, adrenaline, and serotonin, (2) drugs (nonsteroidal anti-inflammatory, anticancer, and antivirals drugs), as well as (3) typical substrate for OATs, such as *para*-aminohippurate (PAH), and (4) specific substrate for Oat3, estrone sulfate (ES) [18]. Previous studies have shown that the differences of organic anion transporters (OATs) functions could be regulated during transcriptional and translational regulations by several compounds. For example, it was found that the phorbol-12-myristate-13-acetate (PMA), a PKC activator, downregulated organic anion transporter 1 (Oat1) and Oat3 functions by decreasing their expression at the plasma membrane [19, 20]. In contrast, insulin and epidermal growth factor (EGF) upregulated Oat1 and Oat3 by increasing their expression at the plasma membrane [21]. Besides these, pathological status also specifically influenced OATs expressions and functions and subsequently altered the pharmacokinetics of their substrates. These consequences might also potentially exert renal toxicity or dysfunction. For instance, previous study reported that streptozotocin- (STZ-) induced diabetic rats exhibited a decreased expression and function of rat renal organic cation transporters 1 and 2 (rOct1 and 2), resulting in decreased organic cationic clearance [22]. Likewise, our recent study demonstrated the reduction of membrane expression of mouse organic anion transporter 3 (mOat3), but not mOat1, in experimental diabetic model [23], together with rOat3 [24]. Therefore, this study was to evaluate the hypothesis that SNE-rich antioxidant activity protects renal oxidative stress and conserves renal transport function, particularly through rOat3, in T2DM rats. 

## 2. Materials and Methods

### 2.1. Chemicals

Polyclonal rabbit anti-rOat3 antibody was obtained from Cosmobio (Tokyo, Japan). Polyclonal rabbit anti-PKC*α*, phosphorylated PKC*α* (p-PKC*α*), p65 subunit of NF*κ*B (p65 NF*κ*B), goat-anti mouse, and rabbit IgG horseradish peroxidase-conjugated secondary antibodies were purchased from Santa Cruz (Santa Cruz, CA, USA). Monoclonal mouse anti-lamin B1 and polyclonal rabbit anti-phosphorylated PKC*ζ* (p-PKC*ζ*) were purchased from Cell signaling (Danvers, MA, USA). Polyclonal rabbit anti-PKC*ζ* was obtained from Zymed (Invitrogen, Carlsbad, CA, USA). Monoclonal mouse anti-*β* actin was purchased from Abcam (Cambridge, MA, USA). Monoclonal mouse anti-Na^+^-K^+^-ATPase was obtained from Novus biological (Littleton, CO, USA). [^3^H]ES (specific activity; SA 50 Ci/mmol) and [^3^H]PAH (SA 1 Ci/mmol) were purchased from Perkin Elmer (Waltham, MA, USA). Unlabeled ES, PAH, streptozotocin (STZ), and CelLytic MT mammalian tissue lysis/extraction reagent were purchased from Sigma Aldrich (Saint Louise, MO, USA). Ascorbic acid was obtained from Merck (Damstadt, Germany), insulin was obtained from Biocon (Bangkok, Thailand), and complete protease inhibitor cocktail was purchased from Roche Applied Science (Indianapolis, IN, USA). Thiobarbituric acid reactive substances (TBARS) assay was purchased from Cayman Chemical (MI, USA). All other chemicals with high purity were obtained from commercial sources. 

### 2.2. *Spirogyra neglecta* Extract Preparation, Purification, and Qualification

*Spirogyra neglecta *extract was identified for species and kindly provided by Dr. Yuwadee Peerapornpisal, Faculty of Sciences, Chiang Mai University. A voucher specimen (number AARL G047) has been deposited at the herbarium of the Applied Algal Research Laboratory, Department of Biology, Faculty of Science, Chiang Mai University, Chiang Mai, Thailand.

Dried SNE was weighed and blended thoroughly followed by boiling at 100°C for 1 hr. The extract was then filtered through filter paper (Whatman, Kent, UK) with negative pressure pump (Hicovac, Köln, Germany). The filtrate was subsequently evaporated using lyophilizer (GEA process engineering Inc., SC, USA). Lyophilized SNE was stored at 4°C prior subsequent experiments. The SNE was standardized by determination of total phenolic content using Folin-Ciocalteu reagent as was recently described by Rattanapot et al. [25]. The quality was quantified to reach a minimum of 77 mg gallic acid equivalent before use in this study.

### 2.3. Animals and Induction of Experimental Diabetes

Male Wistar rats with 120–150 g were obtained from the National Laboratory Animal Center, Mahidol University, Salaya, Thailand. The animal facilities and protocols were approved by the Laboratory Animal Care and Use Committees at Faculty of Medicine, Chiang Mai University, Chiang Mai, Thailand. All experimental rats were housed in a room and maintained at 25 ± 1°C on a 12 hr light/dark cycle. 

Animals were randomized and equally divided into 7 groups: normal diet (20% fat of total energy) (ND), normal diet supplemented with SNE at the dose of 1.0 g/kg BW (ND + SNE1.0), T2DM (DM), T2DM supplemented with SNE at the dose of 0.25, 0.5, and 1.0 g/kg BW, respectively (DM + SNE0.25, DM + SNE0.5, and DM + SNE1.0), and T2DM supplemented with antioxidant compound, ascorbic acid (vitamin C), at the dose of 0.2 g/kg BW (DM + vitamin C) similar to that of the treatment previously described by Owu et al. [26]. These DM + vitamin C rats were designated as positive control of antioxidant treatment in this study. In normal diet rats, the animals were fed with a standard pellet diet (C.P. Mice Feed; S.W.T. Co., Ltd., Samut Prakan, Thailand). T2DM in rats was induced by a combination of high-fat diet (60% fat of total energy) ad libitum and low-single dose of STZ intraperitoneal injection (40 mg/kg BW) as previously described [27, 28]. Ten days after injection, the rats with fasting blood glucose levels exceeded 250 mg/dL were considered T2DM. SNE or vitamin C was subsequently administered daily by oral gavage feeding for 12 weeks. At the end of study, the animals were sacrificed; blood and tissue samples were collected for subsequent experiments. 

### 2.4. Determination of Plasma Glucose, Triglyceride, and Insulin Levels

To characterize T2DM rat model, the quantitative total plasma glucose and triglyceride were determined by commercial enzymatic colorimetric assays (Biotech, Bangkok, Thailand). The plasma insulin concentration was determined using a Sandwich ELISA assay kit obtained from LINCO research (Millipore, MA, USA). Insulin resistance index was estimated using the homeostatic model assessment of insulin resistance (HOMA index) that was calculated by the following formula: fasting plasma insulin ((*µ*U)/mL) × fasting plasma glucose (mmol/L)/(22.5).

### 2.5. Measurement of Total Malondialdehyde Level in Renal Cortical Tissues

In order to determine renal oxidative stress condition, the measurement of malondialdehyde (MDA) levels in renal cortical tissues was carried out. Briefly, the rats were euthanized by pentobarbital and sacrificed. Kidneys were excised and renal cortical tissues were cut and suspended in CelLytic MT mammalian tissue lysis/extraction reagent (Sigma Aldrich, MO, USA) containing 1% complete protease inhibitor cocktail (Roche Applied Science, IN, USA) according to the manufacturer's protocol. The tissues were then homogenized and centrifuged at 1,600 g for 10 min at 4°C. The supernatants were collected for determination of MDA concentrations using commercial TBARS assay kit purchased from Cayman Chemical (Ann Arbor, MI, USA). Each sample was expressed as total MDA level to total protein concentration (nmol/mg protein). 

### 2.6. Histological Examination

To assess renal morphology, the kidneys were excised and one half of the kidney was fixed in 10% neutral formalin buffer for 12–24 hrs and then embedded in paraffin. Each slide was cut into 5–7 *μ*m thick sections and subsequently stained by hematoxylin and eosin (H&E) and the lesions were confirmed by periodic acid-Schiff base (PAS), which are appropriate standard methods for renal biopsy as previously described [29]. The tissue morphological changes were determined using bright-field microscopic evaluation. The morphological analysis of glomerular size, mesangial matrix, and tubular lesions was semiquantitatively assessed by a modification method of diabetic nephropathy classification previously described by Tervaert et al. [29] and Oh et al. [30]. The severity was graded to mild, moderate, and severe for focal changes with less than 25%, 25–50%, and greater than 50% of lesion, respectively. 

### 2.7. Subcellular Fractionation and Western Blot Analysis

To quantitate target protein expressions in each cellular compartment, whole cell lysate, membrane, cytosolic, and nuclei fractions were prepared using differential centrifugation as we previously described [24]. Briefly, renal cortical tissues were cut, suspended in CelLytic MT mammalian tissue lysis/extraction reagent (Sigma Aldrich, MO, USA) containing 1% complete protease inhibitor cocktail (Roche Applied Science, IN, USA) according to the manufacturer's protocol, and subsequently centrifuged at 5,000 g for 10 min at 4°C. The supernatant was designated as *whole cell lysate* and the pellet was resuspended in the same solution and centrifuged at 10,000 g for 10 min at 4°C. The supernatant was then designated as *nuclei-enriched fraction*. In addition, whole cell lysate fraction was subsequently centrifuged at 100,000 g for 2 hrs at 4°C. The supernatant fraction from the spin was designated as the *cytosolic fraction*. The crude membrane pellets were resuspended in the same reagent and designated as *membrane fraction*. The total protein concentration of the samples was measured using Bradford assay (Bio-Rad, CA, USA) and stored at −80°C prior subsequent experiments. 

For western blot analysis, the protein samples (50 *μ*g/lane for membrane and cytosolic samples and 100 *μ*g/lane for whole cell lysate and nuclei samples) were heated in 2X Laemmli solution and separated in 10% sodium dodecyl sulfate polyacrylamide gel. The proteins were then transferred into polyvinylidene difluoride membranes (PVDF) (Millipore, MA, USA) using the Bio-Rad system (Bio-Rad, CA, USA). Nonspecific bindings on the membrane were blocked by 5% nonfat dry milk in Tris-buffer saline (TBS) containing 0.1% tween 20 (TBST) solution for 1 hr at 4°C and incubated overnight with desired primary antibody against rOat3, PKC*α*, p-PKC*α*, PKC*ζ*, p-PKC*ζ*, and p65NF*κ*B (see figure legends in detail). To confirm the enrichment of the fraction, anti-Na^+^-K^+^-ATPase and anti-lamin B1 antibodies were used as membrane and nuclei fractions, respectively, similarly to previously described [31, 32]. Anti-*β* actin antibody was used as loading control for all samples. PVDF membranes were subsequently washed with TBST and incubated with goat-anti mouse or rabbit IgG horseradish peroxidase-conjugated secondary antibody (Santa Cruz, CA, USA) for 1 hr at 4°C. Enhanced chemiluminescent kit (GE Health Care, Buckinghamshire, UK) was utilized to detect the target proteins. The band density was quantitatively analyzed by Image J program from Research Services Branch (RSB) of the National Institute of Mental Health (NIMH, MD, USA). 

### 2.8. Quantitative Real-Time PCR Analysis

Total RNA was extracted and isolated from renal cortex using RNA extraction kit (Amresco, OH, USA), according to the manufacturer's instruction. The first-strand complementary DNA (cDNA) was synthesized using iScript cDNA synthesis kit (Bio-rad, CA, USA) and quantitative real-time PCR (qPCR) was performed using Bio-Rad iQ SYBR green supermix on Bio-Rad iQ5 (Bio-Rad, CA, USA). Primers were designed according to published sequences (Table 1) and were purchased from Integrated DNA technologies (Coralville, IA, USA). Gene expressions were normalized to *β*-actin mRNA levels and reported as relative fold changes (RFC). QPCR amplification was performed in duplicates for each cDNA.

### 2.9. Renal Slice Preparation and Transport Study

It has been suggested that Oat3 is highly expressed in kidneys, liver, and choroid plexus and has a major impact on the secretion and excretion of anionic drugs and xenobiotics for maintaining systemic homeostasis [18]. These organic anions include PAH, prototype substrate for organic anion transporters (OATs), and ES, that is known as the specific substrate for only Oat3 [17]. To determine secretory function of the kidney, organic anions (PAH and ES) uptake mediated by Oat3 was performed using renal cortical slices as previously described [21]. Briefly, after the kidneys were removed and placed in oxygenated saline buffer, renal cortical slices (≤0.5 mm; 5–15 mg, wet weight) were cut with a Stadie-Riggs microtome and maintained in ice-cold oxygenated modified Cross and Taggart buffer containing (mM): 95 NaCl, 80 mannitol, 5 KCl, 0.74 CaCl_2_, and 9.5 Na_2_HPO_4_, pH 7.4. The slices were then incubated in 0.25 mL of buffer containing either 50 nM [^3^H]ES or 5 *μ*M [^3^H]PAH for 30 min at room temperature. For upregulation of rOat3 function by insulin stimulation, the slices were preincubated in 0.5 mL of buffer in the absence or presence of 30 *μ*g/mL insulin for 30 min and subsequently incubated in 0.25 mL of buffer containing either 50 nM [^3^H]ES or 5 *μ*M [^3^H]PAH for 30 min at room temperature. Uptake was stopped by the addition of ice-cold buffer. Slices were rinsed, blotted, weighed, and dissolved in 0.5 mL of 1 N NaOH and neutralized with 0.5 mL of 1 N HCl. The radioactivity was measured using a liquid scintillation analyzer (Perkin Elmer, MA, USA). Transport of ES and PAH was calculated as tissue to medium (T/M) ratio (dpm/g tissue ÷ dpm/mL medium). 

### 2.10. Statistical Analysis

Data were expressed as mean ± S.E.M. Statistical differences were assessed using one-way analysis of variance, followed by Tukey-Kramer test. Insulin stimulation experiment was analyzed using unpaired, two-tailed Student's *t*-test. Statistical analyses were conducted using Statistical Package for the Social Sciences (SPSS) version 11.5 (SPSS Inc., IL, USA). Differences were considered to be significant when *P* < 0.05. 

## 3. Results

### 3.1. Effects of *Spirogyra neglecta* Extract on Type 2 Diabetic Characteristics

 As it was suggested that SNE has antidiabetic effects in T2DM rat model [5], the effects of SNE on general characteristics of T2DM experimental rats in this study were confirmed. As shown in Table 2, after 12 weeks of supplementation, the body weight (BW) and the kidney weight per body weight ratio (KW/BW) were not different among experimental groups. Although there was no significant difference in plasma insulin, the levels of fasting plasma glucose, triglyceride, and HOMA index had significantly increased in T2DM, DM + SNE0.25, and DM + vitamin C rats compared to that of control. These plasma parameters were markedly reduced in DM + SNE0.5 and DM + SNE1.0 rats. This result indicated that SNE was able to reduce blood glucose, triglyceride, and restored insulin resistance after diabetic condition. In addition, the changes in plasma parameters were not observed in ND + SNE1.0, suggesting that SNE at the dose of 1.0 g/kg BW/day did not have any effect on plasma glucose and lipid levels in normal rats.

### 3.2. Effects of *Spirogyra neglecta* Extract on Kidney Morphology in T2DM Rats

**Figure 1:**
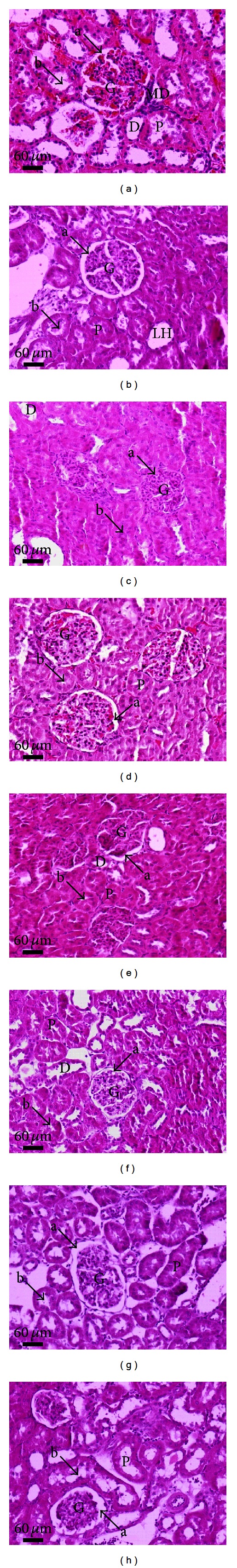
Micrographs of conventional hematoxylin and eosin (H&E) staining of rat kidneys. A sagittal half of kidney from each experimental group was removed, fixed, embedded, cut, and stained by H&E (original magnification 200x for all panels). The data were repeated at least 3 times from separate sets of animals. The results were analyzed using bright-field microscopy. (a) Normal (ND), (b) normal treated with SNE (ND + SNE1.0), (c) T2DM (DM), (d) T2DM treated with vitamin C (DM + vitamin C), (e) T2DM treated with SNE at the dose of 0.25 g/kg BW (DM + SNE0.25), (f) T2DM treated with SNE at the dose of 0.5 g/kg BW (DM + SNE0.5), and (g and h) T2DM treated with SNE at the dose of 1.0 g/kg BW (DM + SNE1.0). Arrow “a” in all panels indicates the Bowman's capsule space. Arrow labeled “b” indicates the proximal tubular epithelial cells and the lumen space. Abbreviation: glomerulus (G), Bowman's capsule (Bm), macular densa (MD), proximal convoluted tubule (P), loop of Henle (LH), and distal convoluted tubules (D).

**Figure 2:**
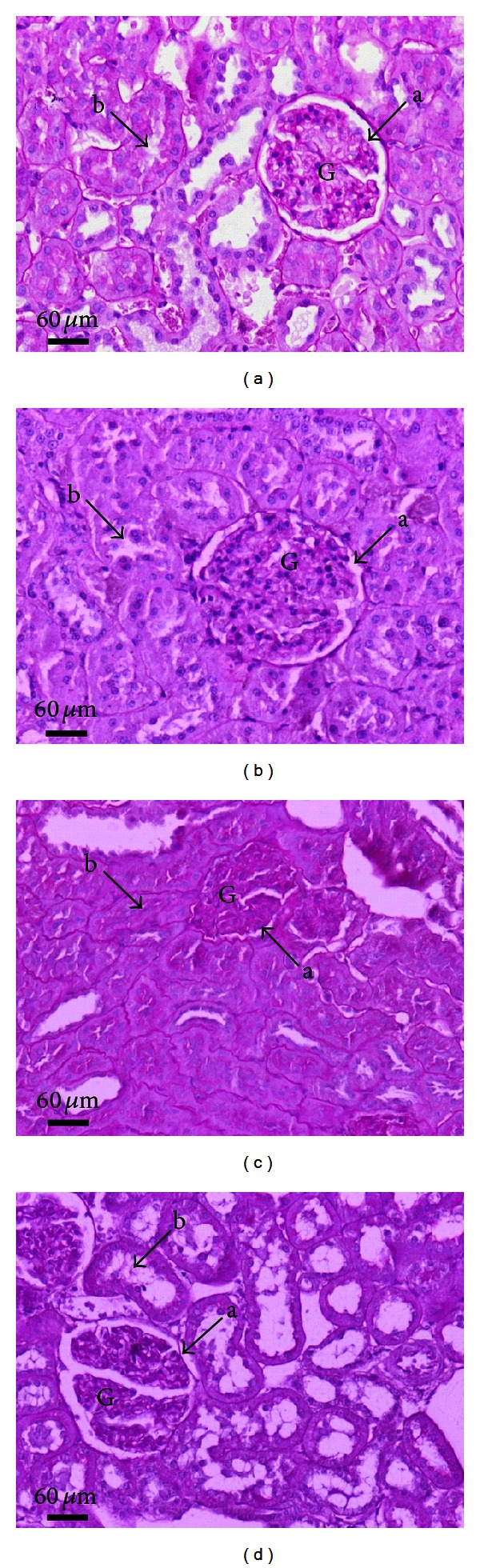
Micrographs of periodic acid-Schiff (PAS) staining of rat kidneys. Light microscopy of sagittal half of kidney sections stained with PAS and counterstained with hematoxylin is shown (original magnification 200x for all panels). (a) Normal (ND), (b) T2DM treated with vitamin C (DM + vitamin C), (c) T2DM (DM), and (d) T2DM treated with SNE at the dose of 1.0 g/kg BW (DM + SNE1.0). The lesions of mesangial cells and glomerulus are indicated by arrow “a”; the proximal tubular epithelium and the lumen are indicated by arrow “b.” Abbreviation: glomerulus (G).

To determine the effect of SNE on renal structural changes in T2DM rat model, renal morphological analysis was semiquantitatively assessed using standard methods for renal biopsy, hematoxylin and eosin (H&E; Figure 1), and periodic acid-Schiff base (PAS; Figure 2) stains. As shown in Figure 1(a), normal diet-fed rats (ND) had normal renal structures including glomerulus, proximal convoluted tubule, and distal convoluted tubule connected with macular densa which was adjusted next to the vascular pole similar to that of ND + SNE1.0 rats (Figure 1(b)). Moreover, T2DM rat kidney markedly exhibited the glomerular hypertrophy, thickening of the mesangium, and glomerular basement membrane (Figure 1(c)). Due to glomerular hypertrophic formation, T2DM rat kidney had shown no Bowman's capsular spaces, together with a contraction of luminal spaces (Figure 1(c), arrow a). This hypertrophy was also present in proximal convoluted tubules (P) (Figure 1(c), arrow b) whereas distal convoluted tubules (D in Figure 1(c)) showed less hypertrophic appearance. Although glomerular hypertrophy was not observed in DM + vitamin C (Figure 1(d) arrow a), a narrow of the renal tubular space of this group was observed (Figure 1(d), arrow b), implying that vitamin C did not fully improve renal structures after DM condition. Similarly to T2DM, glomerular capillaries and tubular cells of DM + SNE0.25 and DM + SNE0.5 persistently increased in sizes (Figures 1(e) and 1(f), resp.). Interestingly, the morphometric analysis showed a significant decrease in both mesangial and tubular hypertrophy in renal tissues of DM + SNE1.0 (Figures 1(g) and 1(h), arrows a and b). In the PAS staining, the results showed moderate lesion of mesangium, glomerular basement membrane, and a narrow of renal tubular lumen in T2DM when compared to that of ND and DM + vitamin C (Figure 2(c) versus Figures 2(a) and 2(b)). Surprisingly, the kidney morphology of DM + SNE1.0 was improved similarly to that of ND, including the reduction in the extent of the glomerular hypertrophy and mesangial expansion (Figure 2(d)). As a result, the tubular lumen and Bowman's capsule spaces were seen (Figure 2(d) arrows a and b). Together, this data clearly demonstrated that SNE at the dose of 1.0 g/kg BW was able to reduce the morphologically deleterious effect of the kidney in T2DM rats without detrimental effect of the kidney in normal rats. 

### 3.3. Effect of *Spirogyra neglecta* Extract on Renal Cortical MDA Concentration

**Figure 3:**
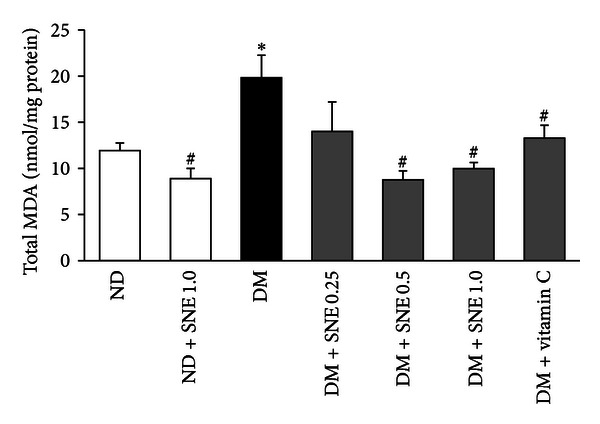
Effect of *Spirogyra neglecta* extract on renal cortical MDA concentrations. Malondialdehyde concentrations (MDA) were determined in renal cortical tissue homogenates from each experimental group using commercial TBARS assay kit. The intensities were read using the absorbance colorimetric method at the wavelength of 540 nm. The results are expressed as mean ± S.E.M. (*n* = 6). *P < 0.05 indicates the significant differences from normal (ND) and ^#^ *P* < 0.05 indicates the significant differences from T2DM (DM) rats.

To determine renal oxidative stress and evaluate antioxidant effect of SNE, the total MDA level was determined in renal cortical tissues using TBARS assay. As shown in Figure 3, T2DM and DM + SNE0.25 renal cortical tissues had a significant increase in renal lipid peroxidation as indicated by high levels of MDA compared to that of control. In contrast, total MDA was decreased in DM + SNE0.5 and DM + SNE1.0, similarly to that of DM + vitamin C. In addition, SNE did not alter MDA level in normal rats as shown in ND + SNE1.0, suggesting that SNE at the dose of 1.0 g/kg BW did not produce lipid oxidation in rat renal tissues. 

### 3.4. Effects of *Spirogyra neglecta* Extract on ES and PAH Transport Mediated by rOat3

**Figure 4:**
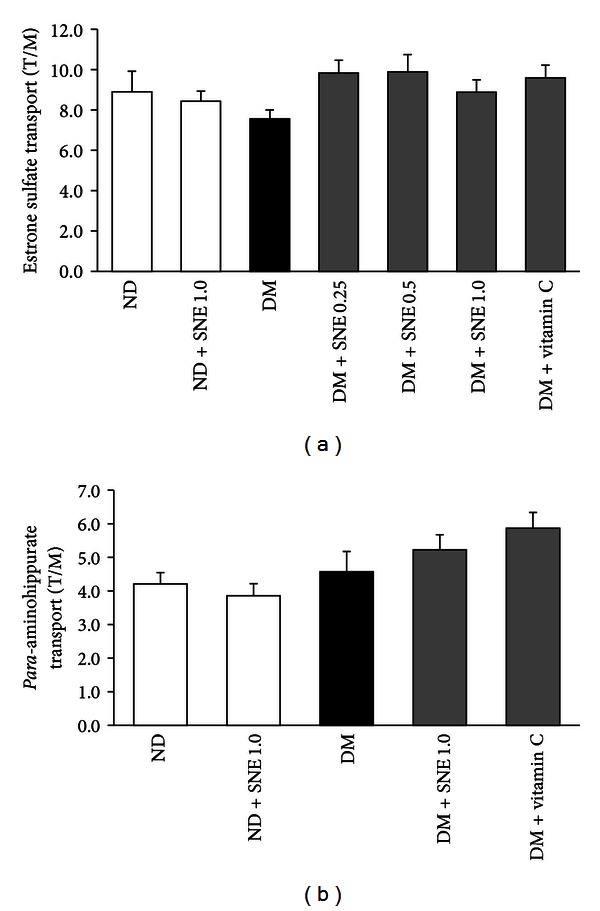
Effects of *Spirogyra neglecta* extract on renal rOat3 substrate transports. Rat renal cortical slices from each experimental groups were incubated for 30 min at room temperature with organic anion substrates, (a) 50 nM of [^3^H]ES or (b) 5 uM of [^3^H]PAH. Data are expressed as tissue to medium ratios (T/M), that is, tissue content (DPM/g) ÷ medium (DPM/mL). The results are expressed as mean ± S.E.M. Each experiment was performed from separate sets of animals using 5 slices from each animal (*n* = 6).

As mentioned above, Oat3 appears to be a primary OAT contributing to cellular transport of organic anions in secretory tissues including kidney, liver, and choroid plexus [18]. Thus, we focused and conducted functional Oat3 transport using both [^3^H]ES, specific substrate of Oat3, and [^3^H]PAH, a typical substrate of OATs, in renal cortical slices. As shown in Figure 4(a), [^3^H]ES uptake mediated by rOat3 was not significantly different among experimental groups. The similar result was also seen in rOat3 transport mediated by [^3^H]PAH uptake (Figure 4(b)). This result indicated that this experimental T2DM condition did not affect physiological rOat3 function. Moreover, SNE concentrations up to 1.0 g/kg BW/day did not change renal rOat3 transport function.

**Figure 5:**
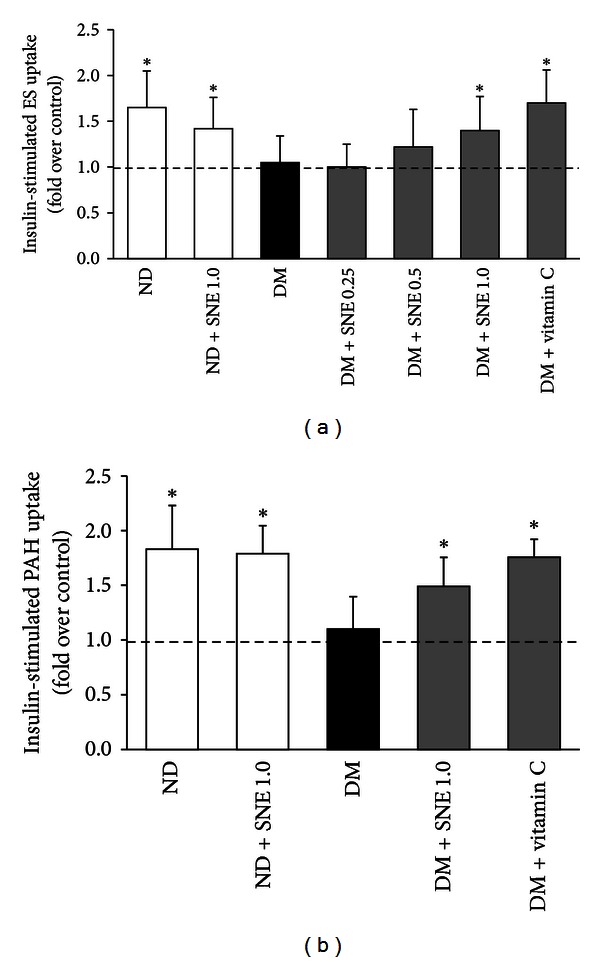
Effects of *Spirogyra neglecta* extract on insulin-stimulated rOat3 substrate transports. Rat renal cortical slices were preincubated for 30 min in the presence or absence of 30 *μ*g/mL insulin and followed by incubation with organic anion substrates. (a) 50 nM of [^3^H]ES or (b) 5 *μ*M of [^3^H]PAH for 30 min. Data are expressed as fold over control (without insulin). Each experiment was performed from separate animals and at least 5 renal slices were used in each condition (*n* = 6). *P < 0.05 indicates significant differences from the slices incubated with buffer alone.

We further addressed whether SNE had any effect on the regulatory mechanism of rOat3 function. A study by Barros et al. [21] had shown that rOat3 function was upregulated after insulin preincubation. We, therefore, determined the regulatory function of rOat3 using both [^3^H]ES and [^3^H]PAH uptakes in rat renal cortical slices in the presence or absence of insulin preincubation. As shown in Figure 5, the slices from control rats that were preincubated with 30 *μ*g/mL of insulin had significant increases in both [^3^H]ES (Figure 5(a)) and [^3^H]PAH (Figure 5(b)) uptakes compared to the slices without insulin, indicating that upregulated rOat3 function by either ES or PAH was reproduced similarly to that of previous study [27]. On the other hand, the effect of insulin on [^3^H]ES uptakes was abolished in renal slices of T2DM, DM + SNE0.25, and DM + SNE0.5 rats. Similar to ES transport, insulin stimulation did not increase [^3^H]PAH uptake in T2DM, suggesting that T2DM and DM treated with low doses of SN extract had significantly impaired insulin-stimulated rOat3 function. Interestingly, the effect of insulin stimulation on the increase of both ES and PAH substrate transports remained present in the renal slices from ND + SNE1.0, DM + SNE1.0, and DM + vitamin C similar to the slices from control, implying that 1 g/kg BW of SNE was able to improve regulatory function of rOat3. 

### 3.5. *Spirogyra neglecta* Extract Did Not Alter rOat3 Expression

**Figure 6:**
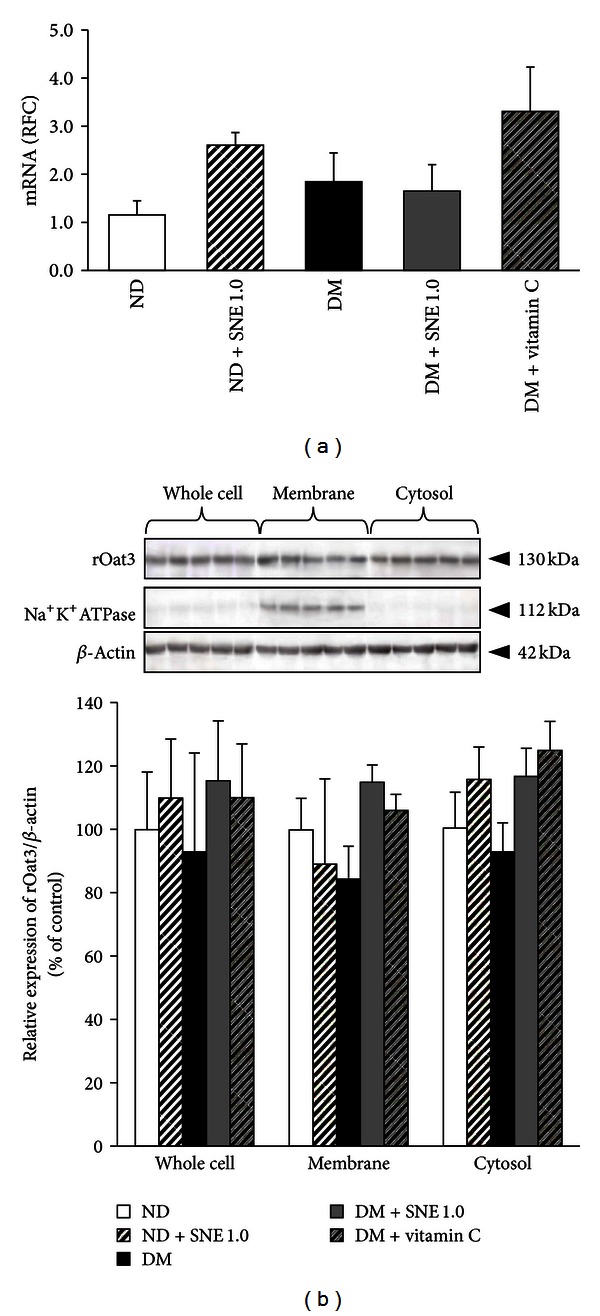
Renal rOat3 mRNA and protein expressions. (a) mRNA expressions of rOat3 from ND, ND + SNE1.0, DM, DM + SNE1.0, and DM + vitamin C rat kidneys. Total RNA was extracted from rat cortical tissues and mRNA levels were measured using quantitative real-time PCR (qPCR). The data are expressed as mean ± S.E.M. and repeated from separate animals (*n* = 6). (b) Western blot analysis of rOat3 protein expression in subcellular fractions from rat renal cortex. Whole cell lysate, membrane, and cytosolic fractions were extracted from rat renal cortical tissues. The samples were then separated using electrophoresis and western blotting. Anti-rOat3 antibody was subsequently detected whereas anti-Na^+^-K^+^-ATPase and anti-*β*-actin antibodies were also used as a membrane marker and loading control, respectively. The data are expressed as mean ± S.E.M. and repeated from separate sets of animals (*n* = 6). A representative blot of rOat3 protein expression is shown in (top (b)) and quantification of relative rOat3/*β*-actin protein expression in each fraction is presented in (bottom (b)).

To further investigate the mechanism by which SNE improved rOat3 upregulation, we primarily postulated that SNE might induce rOat3 expression, leading to rOat3 upregulation in T2DM rats. Thus, rOat3 expressions were subsequently analyzed using qPCR and western blotting as described above in each respective materials and methods section. As shown in Figure 6(a), mRNA levels of rOat3 were not significantly different among experimental groups. Since the uptake profiles of the shared Oat1/Oat3 substrate, PAH, were very similar to that of ES uptake (Figure 5(b)), we also determined rOat1 mRNA expression. Likewise, rOat1 mRNA expression was not significantly different among experimental groups (data not shown). The detection of rOat3 protein expressions was subsequently quantitated in whole cell lysate, membrane, and cytosolic fractions by densitometry. The amount of rOat3 expression in each sample was then normalized by the amount of *β*-actin present in each respective fraction as shown in Figure 6(b). Consistent with mRNA expression, 130 kDa of the rOat3 protein expression in whole cells, membrane, and cytosolic fractions was not significantly different among experimental groups, whereas the anti-Na^+^K^+^-ATPase antibody was detected in only membrane samples of each experimental group. Therefore, this result indicated that improved upregulation of rOat3 function in T2DM by SNE was not caused by increased Oat3 expression but probably involved with other mechanisms. 

### 3.6. *Spirogyra neglecta* Extract Restored Regulatory Function of rOat3 through Stress-Sensitive Pathway

**Figure 7:**
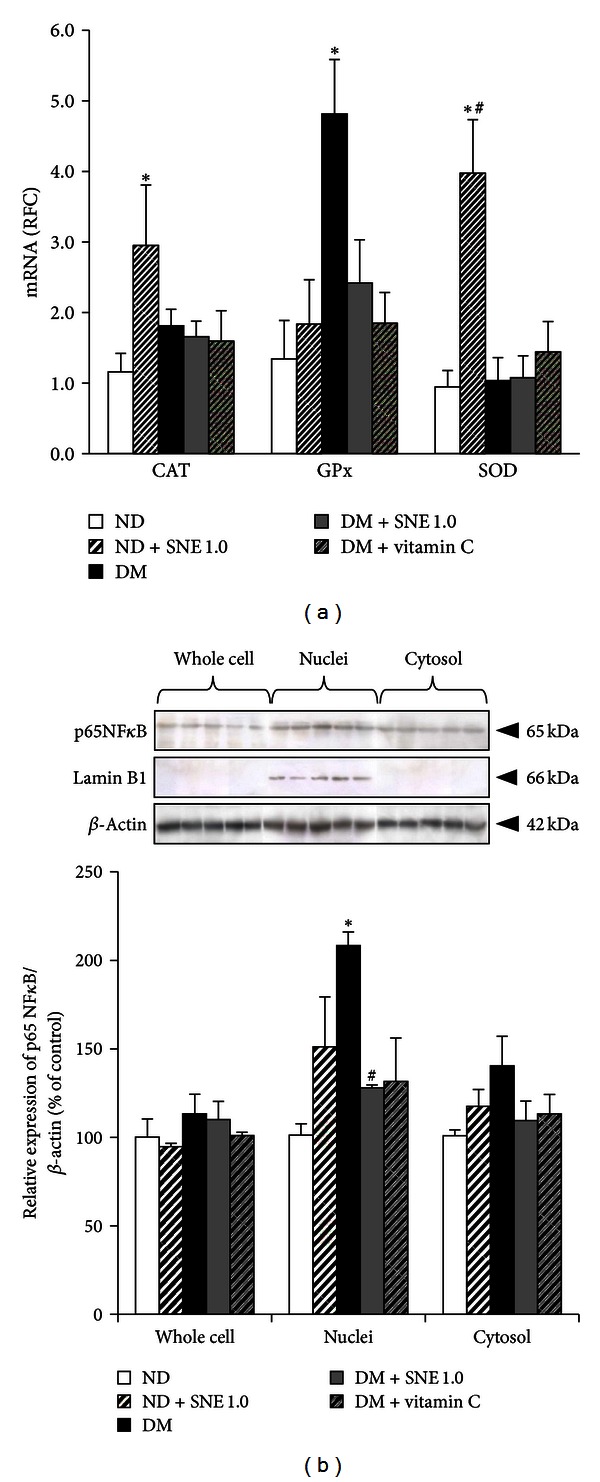
Effect of *Spirogyra neglecta* extract on the expressions of stress-sensitive genes. (a) mRNA expression levels of catalase (CAT), glutathione peroxidase (GPx), and copper-zinc superoxide dismutase (Cu-ZnSOD) from ND, ND + SNE1.0, DM, DM + SNE1.0, and DM + vitamin C  rat kidneys. Total RNA was extracted from rat cortical tissues and mRNA levels were measured using quantitative real-time PCR (qPCR). The results are expressed as mean ± S.E.M (*n* = 6). (b) p65NF*κ*B expression in subcellular fractions of rat kidneys. Whole cell lysate, cytosolic, and nuclei fractions were extracted from rat renal cortical tissues. The samples were then separated using electrophoresis and western blotting. Anti-p65NF*κ*B antibody was subsequently detected while anti-lamin B1 and anti-*β*-actin antibodies were used as a nuclei marker and loading control, respectively. The data are expressed as mean ± S.E.M. and repeated from separate sets of animals (*n* = 6). A representative blot of p65 NF*κ*B protein expression is shown in (top (b)) and quantification of relative protein expression in each fraction is presented in (bottom (b)). *P < 0.05 indicates the significant differences from normal (ND) and ^#^ *P* < 0.05 indicates the significant differences from T2DM (DM) rats.

Since the earlier experiment has shown that the effect of SNE was not involved with rOat3 expression and the study by Peerapornpisal et al. [4] demonstrated an antioxidant activity of SNE *in vivo*, thus, we subsequently analyzed the oxidative stress markers that have been proposed to be the underlying factors of the development of diabetic nephropathy as previously described [9]. These included three major free radical scavenger enzymes (catalase, CAT; glutathione peroxidase, GPx; Cu-Zn superoxide dismutase, Cu-ZnSOD) and a common stress response gene, nuclear factor *κ*-B (NF*κ*B). These enzymes and nuclear factor were quantitatively analyzed using qPCR and western blotting, respectively. As shown in Figure 7(a), T2DM rat kidneys showed a significant increase in the expression of free radical scavenger transcript, GPx, compared to that of control. In contrast, DM + SNE1.0 and DM + vitamin C showed substantially reduced GPx expression relative to that of T2DM. This data indicates a presence of high oxidative stress condition in T2DM rat model, reflected by an increased GPx expression against ROS. Our data also showed that SNE had substantially reduced GPx expression similar to that of vitamin C. In addition, ND + SNE1.0 was significantly increased in CAT and Cu-ZnSOD mRNA expressions, suggesting that SNE had antioxidant effect by inducing antioxidant enzyme transcriptions. 

Increased NF*κ*B has been proposed to be a common stress response factor under hyperglycemia, elevated free fatty acid (FFA), and oxidative stress [9]. During resting state, inactive heterodimers of NF*κ*B is present in the cytosol. Once it is activated by either hyperglycemia, elevated FFA, or ROS, p65 subunit of NF*κ*B (p65NF*κ*B) would be translocated into the nucleus [9, 33]. Such findings led us to quantify the amount of p65NF*κ*B expression in subcellular fractions extracted from renal cortical tissues using western blotting analysis. The detection of p65NF*κ*B bands was analyzed by densitometry. The amount of p65NF*κ*B expression in each sample was normalized by the amount of *β*-actin present in each respective fraction as shown in Figure 7(b). Consistently, the primary antibody against lamin B1 was detected in only nuclei samples of each experimental group as previously seen [31]. Furthermore, there was no significant difference in p65NF*κ*B expression in whole cell and cytosolic fractions among experimental groups. However, NF*κ*B activation (p65NF*κ*B) was significantly increased in nuclei fraction of T2DM compared to that of control, whereas this activation was markedly decreased in DM + SNE1.0 relative to T2DM rat kidneys. This result indicated that SNE improved oxidative stress in T2DM by preventing the activation and translocation of NF*κ*B. 

### 3.7. *Spirogyra neglecta* Extract Modulated rOat3 Function Through Protein Kinases

**Figure 8:**
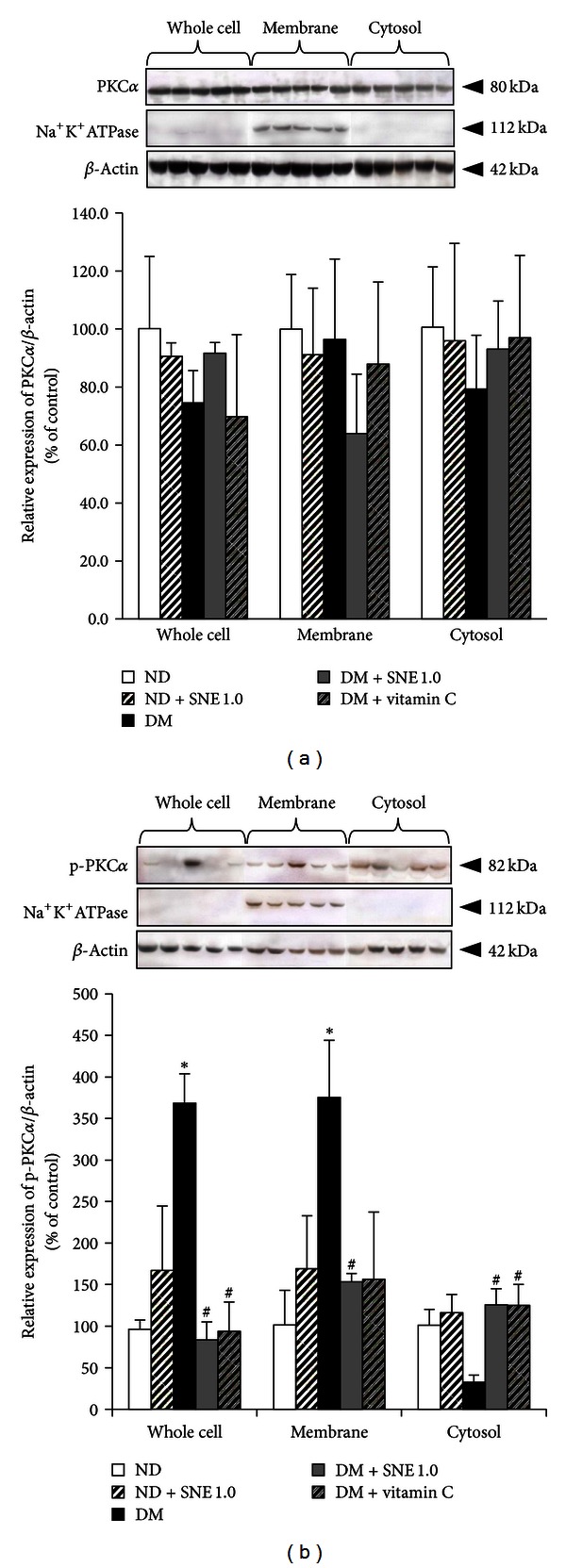
Effects of *Spirogyra neglecta* extract on expression of PKC*α* and p-PKC*α* in renal cortical tissues. (a) Western blotting of whole cell lysate, membrane, and cytosolic fractions from rat kidneys was separated and detected using (a) anti-PKC*α* and (b) anti-p-PKC*α* antibodies from ND, ND + SNE1.0, DM, DM + SNE1.0, and DM + vitamin C rat kidney cortex. Anti-Na^+^-K^+^-ATPase and anti-*β*-actin antibodies were used as a membrane marker and loading control, respectively. Densitometry was analyzed and expressed as mean ± S.E.M. (*n* = 6). *P < 0.05 indicates the significant differences from normal (ND) and ^#^ *P* < 0.05 indicates the significant differences from T2DM (DM) rats. A representative blot of PKC*ζ*/*β*-actin or p-PKC*ζ*/*β*-actin ratios is shown in (top (a, b)) and quantification of relative protein expression in each fraction is presented in (bottom (a, b)).

**Figure 9:**
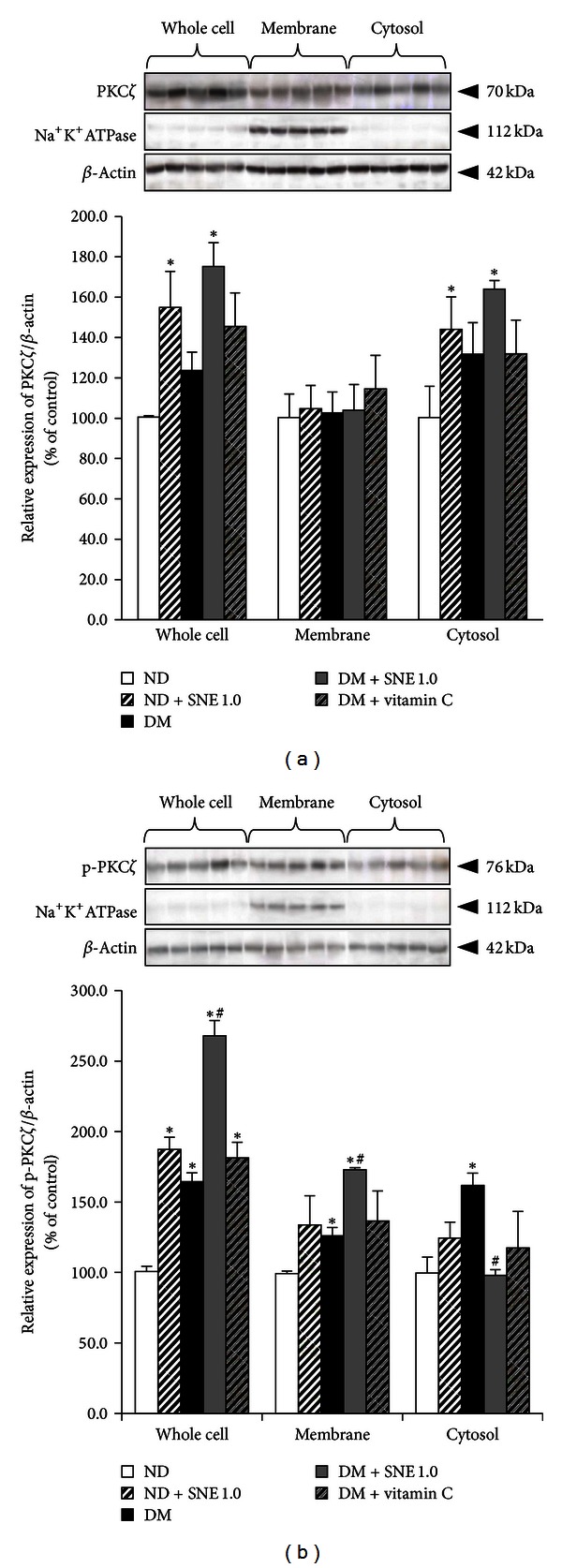
Effects of *Spirogyra neglecta* extract on expression of PKC*ζ* and p-PKC*ζ* in renal cortical tissues. (a) Western blotting of whole cell lysate, membrane, and cytosolic fractions from rat kidneys was separated and detected using (a) anti-PKC*ζ* and (b) anti-p-PKC*ζ* antibodies from ND, ND + SNE1.0, DM, DM + SNE1.0, and DM + vitamin C rat kidney cortex. Anti-Na^+^-K^+^-ATPase and anti-*β*-actin antibodies were used as a membrane marker and loading control, respectively. Densitometry was analyzed and expressed as mean ± S.E.M. (*n* = 6). *P < 0.05 indicates the significant differences from normal (ND) and ^#^ *P* < 0.05 indicates the significant differences from T2DM (DM) rats. A representative blot of PKC*ζ*/*β*-actin or p-PKC*ζ*/*β*-actin ratios is shown in (top (a, b)) and quantification of relative protein expression in each fraction is presented in (bottom (a, b)).

Since our data demonstrated that the effect of SNE has improved the regulatory function of rOat3 (Figure 5), and that it at least partly linked to stress-sensitive signaling pathway (Figure 7), we then further addressed whether such effect could be also involved with rOat3 signaling pathway. Previous report suggested that the PKC*α* activation downregulated Oat3 function [34]. In addition, our recent study also demonstrated that phosphorylated PKC*α* (p-PKC*α*) increased at the plasma membrane of renal cortical tissues in STZ-induced diabetic rats when compared with normal rats [24], whereas Barros et al. [21] had previously shown that upregulated rOat3 function by insulin or EGF shared a common pathway through PKC*ζ* activation. The latter study, however, investigated only PKC*ζ* activity and had not referred to its localization after activation. Therefore, whether SNE has any effect on specific protein kinases, PKC*α* and PKC*ζ*, regulated rOat3 function in T2DM was identified in subcellular fractions extracted from renal cortical tissues using western blotting analysis. The detection of PKC*α*, p-PKC*α*, PKC*ζ*, and p-PKC*ζ* proteins was analyzed by densitometry and the amount of each target protein in each sample was normalized by the amount of *β*-actin present in each respective fraction as shown in Figures 8–9. Consistently, Na^+^K^+^-ATPase was detected in only membrane samples of each experimental group. As shown in Figure 8(a), total PKC*α* was not significantly different in whole cell lysate, membrane, and cytosolic fractions among experimental groups. However, activated PKC*α* (p-PKC*α*) was markedly increased in both whole cell and membrane fractions in T2DM rat kidneys while it was significantly reduced in cytosolic fraction, indicating that oxidative stress in T2DM resulted in the activation and translocation of PKC*α* to the plasma membrane (Figure 8(b)). Interestingly, p-PKC*α* was significantly decreased in both whole cell and membrane fractions in DM + SNE1.0 and DM + vitamin C rat kidneys relative to that of T2DM, suggesting that SNE and vitamin C prevented PKC*α* activation and translocation in T2DM (Figure 8(b)). Nonetheless, p-PKC*α* was not significantly different in any fraction of ND + SNE1.0 samples, indicating that SNE did not alter intracellular PKC*α* proteins under normal condition. 

Unlike PKC*α*, total PKC*ζ* was significantly increased in whole cells and cytosolic fraction in ND + SNE1.0 and DM + SNE1.0 groups when compared to that of normal rat kidneys, which strongly indicated that SNE induced PKC*ζ* new protein synthesis (Figure 9(a)). Surprisingly, activated PKC*ζ* (p-PKC*ζ*) in whole cell lysate, membrane, and cytosolic fractions was significantly increased in T2DM relative to control, suggesting that diabetic condition also influenced PKC*ζ* activity (Figure 9(b)). In addition, a whole cell lysate fraction of ND + SNE1.0, DM + SNE1.0, and DM + vitamin C also showed a significantly increase in p-PKC*ζ* when compared to that of control. This indicated that PKC*ζ* activity could be modulated by several factors, including diabetic status and SNE and/or vitamin C supplementations. Interestingly, the increase of p-PKC*ζ* in membrane fraction was shown in DM and DM + SNE1.0 relative to that of control, but its membrane expression was significantly higher in DM + SNE1.0 than in DM alone, implying that SNE exerted additive effect with diabetic status, resulting in exaggerated PKC*ζ* activity. Simultaneously, only p-PKC*ζ* in cytosolic fraction of DM + SNE1.0 was significantly decreased relative to T2DM. This data suggested that SNE stimulated PKC*ζ* and subsequently induced translocation of p-PKC*ζ* into the plasma membrane, leading to improved functional rOa3 transport under T2DM condition (Figure 9(b)). 

## 4. Discussion

*Spirogyra neglecta* (Hassall) Kützing (SN) or commonly known in Thai as “*Tao*” has been widely grown in the Nan River, Northern Thailand. *In vivo* studies indicated that this species has several beneficial effects, including antigastric ulcer, anti-inflammatory, antihyperglycemic, and antihyperlipidemic actions [3, 5]. Previously, antioxidant activity of SN extract (SNE) has been also shown *in vitro* [4] and its phytochemical compositions recently revealed the presence of phenolics, tannins, glycosides, and saponins [35]. These findings suggested that SNE has potential to be developed as nutraceutical products [4, 35]. To assess this possibility, we evaluated the beneficial effects of SNE in experimental T2DM rat model. This study clearly demonstrated that SNE has antidiabetic, antioxidant, and renoprotective effects in T2DM rat model by improving renal oxidative stress and restoring regulatory function of rOat3. These consequences are essential for maintaining the secretion and elimination of endogenous organic anions, drugs, and xenobiotics and ultimately preventing the progression of diabetic complications. 

As shown in Table 2, the significant increase in the levels of fasting plasma glucose, triglyceride, and HOMA index was exhibited in DM rats, suggesting that hyperglycemia, hypertriglyceridemia, and insulin resistance had developed in our experimental rat model, which is similar to that of T2DM as seen in human. We have also shown that a marked increase of hyperglycemia, hypertriglyceridemia, and HOMA index was reduced by the oral administration of 0.5 and 1 g/kg BW of SNE (Table 2). Recently, it was demonstrated that SNE ameliorated hyperglycemia, dyslipidemia and improved the whole body insulin sensitivity in T2DM rats [5]. However, the precise mechanism of antidiabetic effect needs further investigation. Besides SNE, natural supplements such as onion peel, grape seed, and green tea extracts were previously demonstrated to have antihyperglycemia, antihyperlipidemia and improve insulin resistance in experimental diabetic models through different mechanisms. For instance, onion peel extract ameliorated hyperglycemia and insulin resistance in skeletal muscles and also suppressed oxidative stress and inflammation in liver of T2DM rats [27]. In addition, antihyperglycemic effect was also observed in T1DM rats treated with grape-seed-derived procyanidin extract [36]. Furthermore, both onion peel and green tea extracts also had antihyperlipidemic effect, leading to decreased circulating free fatty acids (FFA) and oxidative stress in insulin resistant rats [27, 37]. 

At the present, several studies have suggested the diagnostic protocols for diabetic-induced renal dysfunction [29, 30]. Such pathological classifications allowed us to obtain the prediction of renal injury in our T2DM rat model. As shown in Figures 1 and 2, T2DM rat kidneys exhibited glomerular hypertrophy, mesangial expansion, and a narrow Bowman's capsule/tubular lumen spaces. Dosage-dependent effect of SNE on renal morphological changes was also observed in this study. Similarly, previous study has shown that diabetic nephropathy rats had hypertrophic appearance in both glomerular capillaries and tubular compartments, which resulted in impaired membrane permeability and function, and the pathological changes were reversed by the angiotensin-converting enzyme inhibitor, quinapril [38]. 

Recently, the role of oxidative stress in diabetic complication has been extensively studied [9, 10]. Persistent hyperglycemia was indicated to be a primary factor of high uptake of glucose into brain, red blood cells, and kidney and subsequently induced cellular oxidative stress [10]. Likewise, mice induced with high-fat diet had shown a significantly increased levels of glycated hemoglobin A_1C_, and this factor was positively correlated with the accumulation of MDA levels in cerebral, liver, and kidney tissues [39]. Apparently, a dose-dependent SNE was too able to reduce renal cortical MDA concentrations (Figure 3), suggesting that SNE plays an important role to attenuate oxidative stress induced by hyperglycemia and/or hyperlipidemia and could prevent the complication in type 2 diabetes. However, the precise mechanism of compositions in SNE exerting this effect requires further investigation.

As mentioned above, organic anion transporter 3 (Oat3) is a membrane transporter that is highly expressed in the renal tubular epithelium, liver, and choroid plexus, where it plays a crucial role in secretion of anionic drugs and toxins and controlling systemic homeostasis of organic anions. The function of Oat3 could be regulated by several compounds, including hormones, endogenous/exogenous substances, and also by pathological status [18, 40]. Previously, the reduction of renal organic cation transporter 1 (Oct1) and Oct2 expressions and functions in STZ-induced T1DM rats was shown [22]. Moreover, we recently demonstrated that mouse Oat3, but not Oat1, showed the decreased expression and the uptake of fluorescein into isolated renal proximal tubules in experimental diabetes induced by STZ. Although fluorescein is the known substrate of both organic anion transporter 1 (Oat1) and Oat3 [17], we were able to show that only Oat3 expression, but not Oat1, was mostly influenced by diabetic condition [23]. In this study, the significant difference of ES and PAH uptake in renal slices among experimental groups was not observed (Figure 4), and these data were also similar to that of rOat3 mRNA and protein expressions. In contrast, upregulation of rOat3 by insulin stimulation was blunted in T2DM and SNE administration was able to restore the insulin effect through increases in both ES and PAH uptakes (Figure 5). Of course, none of these findings preclude the impact of T2DM status on another Oat3 homologue transporter-Oat1. As we expected, there was no difference in rOat1 mRNA expression among experimental groups (data not known). Indeed, the uptake of adeforvir, the specific substrate of basolateral rOat1 [41], and more appropriate model, such as Oat3 knock-out mice, might be more useful tools for differentiation of the impact of T2DM on individual basolateral transporters. More recently, VanWert et al. have reviewed and raised the interesting notion on differential regulation between Oat1 and Oat3 under pathological states [40]. For example, they indicated that as Oat3 transports bile acids, Oat1 does not. The upregulated Oat3 was then seen in cholestasis in rats. Moreover, rOat3 protein was reduced in all cellular fractions of bilateral ureteral obstruction in rats while total rOat1 protein expression was increased. It was also recently found that rOat2 was 2.3-fold overexpressed in T2DM rats when compared with normal, while rOct2 was reduced [42]. In addition, chronic renal failure in rats was shown to correlate with increased rOct2 expression without interfering rOat1, rOat3, and rOct1 expressions [43]. Collectively, it is likely that the pathological condition could differentially regulate individual transporter and any transporter that is influenced by T2DM condition should be precisely investigated. 

We, next, identified the mechanisms by which SNE completely improved upregulation of rOat3 after insulin stimulation. Since SNE enriched polyphenols, which is known to have antioxidant activity [4, 25, 35], we then focused on renal antioxidant system and found that SNE primarily counteracts ROS at the transcriptional process by the alteration of GPx mRNA transcript in DM rats and overexpressed Cu-ZnSOD and CAT mRNA levels in normal rats (Figure 7(a)). In agreement with our data, the expressions and activities of renal GPx and Cu-ZnSOD were also increased in STZ-induced diabetic rats [44] whereas insulin treatment was able to normalize Cu-ZnSOD and CAT mRNA levels [45]. Thus, this finding concluded that the antioxidant activity of SNE could act directly to these free radical scavenging enzymes at their transcriptional modification process and might subsequently modulate other cellular proteins to ameliorate hyperglycemia, hyperlipidemia, insulin resistance, and Oat3 regulatory function. 

Previously, it has been suggested that hyperglycemia-induced ROS production led to renal dysfunction by activating NF*κ*B, a common stress response gene, which, in turn, induced proinflammatory cytokines production including TNF-*α* and IL1-*β* [10, 46, 47]. The significant findings in our study showed that SNE with its polyphenols-rich content strongly inhibited NF*κ*B activation and prevented its translocation into the nucleus (Figure 7(b)); this could lead to the lowering of proinflammatory cytokines production. Simultaneously, this consequence might also modulate phase II antioxidant enzymes, for example, SOD and CAT mRNA expressions. To support this notion, it was recently found that the increased nuclear factor (erythroid-derived 2)-like 2 or Nrf2 protein expression by phenolic acid was positively correlated with the induction of hepatic Cu-ZnSOD, GPx, CAT, and multidrug resistance-associated protein 3 (Mrp3) mRNA expressions [48]. 

Finally, as shown in Figures 8 and 9, we had shown that the specific protein kinase isoform expressions, localizations, and activities were influenced by T2DM status, and the effect of SNE could reverse these observations. As described previously, Oat3 was downregulated by angiotensin II via PKC*α* activation [34] whereas the presence of dynamin was responsible for hOAT1 internalization through clathrin-dependent pathway [49]. Similar to our findings, previous study found that insulin stimulated mouse and rat Oat3 functions through PKC*ζ* activation [21] whereas EGF stimulated Oat3 function through PKA activation [19]. Alternatively, high glucose exposure has been proposed to activate specific PKC*α*, *β*, and *ε* in diabetic nephropathy [50]. Taken together, it is likely that SNE has a direct effect on specific PKC isoforms that mainly regulated rOat3, including PKC*α* and PKC*ζ*, which resulted in retrieving Oat3 function after insulin stimulation in T2DM. 

## 5. Conclusion

We have demonstrated antidiabetic, antioxidant, and renoprotective effects of SNE in long-term hyperglycemia-induced ROS in T2DM experimental rat model. SNE was able to improve hyperglycemia, hypertriglyceridemia, insulin resistance, as well as renal oxidative stress, and regulation of rOat3 transport function. The mechanism by which SNE improved rOat3 regulation was directly linked with the modulation of antioxidant systems including free radical scavenging enzymes and NF*κ*B expression and function. Moreover, our findings indicated that polyphenol-rich SNE acted directly to regulatory proteins of rOat3, PKC*α*, and PKC*ζ*, resulting in changes in their expressions, localizations, and activities. However, further supporting information in experimental or human studies on whether SNE has a distinct effect on particular PKC isoform is needed. The beneficial information of our findings allows for further development of SNE into either nutraceutical or pharmaceutical products for prevention of diabetic nephropathy. 

## Figures and Tables

**Table 1:** Primer sequences and expected amplicon sizes for the gene amplification.

cDNA	Genbank acc. no.	Forward primer (5′ to 3′)	Reverse primer (5′ to 3′)	Amplicon size (bp)
Cu-ZnSOD	X05634	GCAGAAGGCAAGCGGTGAAC	TAGCAGGACAGCAGATGAGT	387
GPx	NM030826	CTCTCCGCGGTGGCACAGT	CCACCACCGGGTCGGACATAC	297
CAT	NM012520	ACAACTCCCAGAAGCCTAAGAATG	GCTTTTCCCTTGGCAGCTATG	76
rOat3	NM031332	ATCTCATCAACATCTATTGGGTACTG	CAGAGAGAGACAGAAGGTCACAC	371
*β*-actin	NM031144	ATGGTGGGTATGGGTCAGAA	GGGGTGTTGAAGGTCTCAAA	241

Cu-ZnSOD; copper-zinc superoxide dismutase, GPx; glutathione peroxidase, CAT; catalase, rOat3; rat organic anion transporter 3.

**Table 2:** Effects of *Spirogyra neglecta* extract on general characteristics of T2DM experimental rats.

	ND	ND + SNE1.0	DM	DM + SNE0.25	DM + SNE0.5	DM + SNE1.0	DM + vitamin C
General characteristic							
BW (g)	516.0 ± 57.3	493.3 ± 40.3	500.0 ± 48.6	510.0 ± 54.5	515.5 ± 49.6	520.0 ± 49.7	525.0 ± 19.1
KW/BW ratio	4.7 ± 0.5	4.9 ± 0.3	5.0 ± 1.1	4.8 ± 0.7	5.0 ± 0.3	4.3 ± 0.9	6.3 ± 1.9

Plasma parameters							
Glucose (mg/dL)	131.0 ± 20.3	124.8 ± 18.3	305.0 ± 58.1*	244.4 ± 58.6*	198.0 ± 14.1^#^	125.6 ± 24.6^#^	277.5 ± 62.5*
Triglyceride (mmol/L)	124.7 ± 11.5	101.6 ± 23.5	266.5 ± 59.3*	229.5 ± 17.5*	145.6 ± 48.2^#^	156.9 ± 34.5^#^	153.5 ± 18.6^#^
Insulin (ng/mL)	2.2 ± 0.5	2.0 ± 0.8	1.7 ± 0.5	1.6 ± 0.1	2.3 ± 1.0	1.0 ± 0.4	1.0 ± 0.6
HOMA index	17.9 ± 4.9	16.0 ± 8.2	31.6 ± 11.4*	28.8 ± 1.9*	16.2 ± 6.1^#^	15.1 ± 7.9^#^	38.4 ± 20.1*

Data are presented as mean ± S.E.M. from 6 animals per group. BW: body weight, KW: kidney weight, HOMA: homeostatic model assessment of insulin resistance, *P < 0.05 indicates the significant differences from normal (ND), and ^#^ *P* < 0.05 indicates the significant differences from T2DM rats.

## Abbreviations

SNE: *Spirogyra neglecta* extract
T2DM: Type 2 diabetic mellitus
Oat1: Organic anion transporter 1
Oat3: Organic anion transporter 3
BW: Body weight
mRNA: Messenger ribonucleic acid
NF*κ*B: Nuclear transcription factor kappa B
p65NF*κ*B: Phosphorylated p65 nuclear factor kappa B
PKC*α*: Protein kinase C alpha
PKC*ζ*: Protein kinase C zeta
ROS: Reactive oxygen species
MAPK: Mitogen-activated protein kinase
AGE: Advanced glycation end-products
PAH: *Para*-aminohippurate
ES: Estrone sulfate
STZ: Streptozotocin
Oct1: Organic cation transporter 1
Oct2: Organic cation transporter 2
mOat3: Mouse organic anion transporter 3
MDA: Malondialdehyde
TBARS: Thiobarbituric acid reactive substances
H&E: Hematoxylin and eosin
PAS: Periodic acid-Schiff base
PVDF: Polyvinylidene difluoride membrane
TBS: Tris-buffer saline
ECL: Enhanced chemiluminescence
PCR: Polymerase chain reaction
cDNA: Complementary deoxyribonucleic acid
qPCR: Quantitative polymerase chain reaction
Cu-ZnSOD: Copper-zinc superoxide dismutase
GPx: Glutathione peroxidase
CAT: Catalase.
